# Effectiveness of polymyxin B-immobilized fiber column in sepsis: a systematic review

**DOI:** 10.1186/cc5780

**Published:** 2007-04-20

**Authors:** Dinna N Cruz, Mark A Perazella, Rinaldo Bellomo, Massimo de Cal, Natalia Polanco, Valentina Corradi, Paolo Lentini, Federico Nalesso, Takuya Ueno, V Marco Ranieri, Claudio Ronco

**Affiliations:** 1 Department of Nephrology, Ospedale San Bortolo, Viale Rodolfi 37, 36100 Vicenza, Italy; 2 Section of Nephrology, Department of Medicine, St. Luke's Medical Center, 279 E Rodriguez Sr Boulevard, Quezon City 1102, Philippines; 3 Section of Nephrology, Department of Medicine, Yale University School of Medicine, 333 Cedar Street FMP 107, New Haven, CT 06520, USA; 4 Department of Intensive Care and Department of Medicine, Austin & Repatriation Medical Centre, Studley Road, Heidelberg, Victoria 3084, Australia; 5 Transplantation Unit, Surgical Services, Massachusetts General Hospital, 55 Fruit Street White 506, Boston, MA 02114, USA; 6 Department of Anesthesia and Intensive Care, Ospedale San Giovanni Battista, Corso Bramante 88, 10126 Torino, Italy

## Abstract

**Introduction:**

Severe sepsis and septic shock are common problems in the intensive care unit and carry a high mortality. Endotoxin, one of the principal components on the outer membrane of gram-negative bacteria, is considered important to their pathogenesis. Polymyxin B bound and immobilized to polystyrene fibers (PMX-F) is a medical device that aims to remove circulating endotoxin by adsorption, theoretically preventing the progression of the biological cascade of sepsis. We performed a systematic review to describe the effect in septic patients of direct hemoperfusion with PMX-F on outcomes of blood pressure, use of vasoactive drugs, oxygenation, and mortality reported in published studies.

**Methods:**

We searched PubMed, the Cochrane Collaboration Database, and bibliographies of retrieved articles and consulted with experts to identify relevant studies. Prospective and retrospective observational studies, pre- and post-intervention design, and randomized controlled trials were included. Three authors reviewed all citations. We identified a total of 28 publications – 9 randomized controlled trials, 7 non-randomized parallel studies, and 12 pre-post design studies – that reported at least one of the specified outcome measures (pooled sample size, 1,425 patients: 978 PMX-F and 447 conventional medical therapy).

**Results:**

Overall, mean arterial pressure (MAP) increased by 19 mm Hg (95% confidence interval [CI], 15 to 22 mm Hg; *p *< 0.001), representing a 26% mean increase in MAP (range, 14% to 42%), whereas dopamine/dobutamine dose decreased by 1.8 μg/kg per minute (95% CI, 0.4 to 3.3 μg/kg per minute; *p *= 0.01) after PMX-F. There was significant intertrial heterogeneity for these outcomes (*p *< 0.001), which became non-significant when analysis was stratified for baseline MAP. The mean arterial partial pressure of oxygen/fraction of inspired oxygen (PaO_2_/FiO_2_) ratio increased by 32 units (95% CI, 23 to 41 units; *p *< 0.001). PMX-F therapy was associated with significantly lower mortality risk (risk ratio, 0.53; 95% CI, 0.43 to 0.65). The trials assessed had suboptimal method quality.

**Conclusion:**

Based on this critical review of the published literature, direct hemoperfusion with PMX-F appears to have favorable effects on MAP, dopamine use, PaO_2_/FiO_2 _ratio, and mortality. However, publication bias and lack of blinding need to be considered. These findings support the need for further rigorous study of this therapy.

## Introduction

Severe sepsis and septic shock are common problems encountered in the intensive care unit (ICU), with an estimated incidence in the United States of 750,000 cases per year and a mortality rate of 25% to 80% [1]. Sepsis involves a complex interaction between bacterial toxins and the host immune system. Bacterial-associated toxins are some of the principal components of gram-negative (endotoxin) and gram-positive (lipotechoic acid) organisms [2-4]. Lipotechoic acid, a product of *Staphylococcal *organisms, promotes production of tumor necrosis factor-alpha (TNF-α) and leads to the development of sepsis and septic shock. Endotoxin, which exists in the outer membrane of gram-negative bacteria, interacts with the host during gram-negative sepsis. Endotoxin causes the release of cytokines such as interleukin (IL)-1 and TNF-α and activates complements and coagulation factors. Endotoxin is considered one of the principal biological substances that cause gram-negative septic shock [2,4]. Nevertheless, anti-endotoxin drug therapies failed to demonstrate a consistent clinical benefit: E5 murine antibody demonstrated non-specific binding/inactivation *in vivo*, conflicting results were seen with HA-1A monoclonal antibody in two separate randomized controlled trials (RCTs), and intravenous polymyxin B has significant nephrotoxic and neurotoxic effects [4-6]. This lack of clinical success with these anti-endotoxin therapies has shifted interest to extracorporeal therapies to reduce circulating levels of endotoxin. Polymyxin B bound and immobilized to polystyrene fibers (PMX-F) has been reported to effectively bind endotoxin in both *in vitro *and *in vivo *studies [7]. The rationale underlying extracorporeal therapy with PMX-F would be to remove circulating endotoxin by adsorption, thus preventing progression of the biological cascade of sepsis. This blood purification medical device has been reimbursed by the Japanese national health insurance program since 1994 [7]. Direct hemoperfusion with PMX-F (DHP-PMX) can be applied to patients with endotoxemia or suspected gram-negative infection who fulfill the conditions of Systemic Inflammatory Response Syndrome and have septic shock requiring vasoactive agents. Since 1994, more than 60,000 patients have received this treatment.

Several studies demonstrate efficient removal of endotoxin with DHP-PMX as well as suppression of *Staphylococcus aureus *lipoteichoic acid-induced TNF-α production [7-24]. However, despite the well-documented capacity to lower blood endotoxin levels, the impact of this therapy on clinical endpoints remains unclear. This systematic review aims to describe the published experience with DHP-PMX as well as the methodological quality of these studies and estimate the magnitude of effect reported in these studies. Because PMX-F does not directly address the source of sepsis, physiologic endpoints such as reduction in vasopressor or ventilatory support, improvement in hemodynamics or oxygenation, and reduction in severity scores, in addition to mortality, are outcomes of clinical interest [25]. Therefore, the primary objective of this systematic review is to describe the effect of PMX-F on blood pressure, use of vasoactive drugs, oxygenation, and mortality. A secondary objective is to describe the effect on endotoxin levels reported in these studies.

## Materials and methods

The search strategy and data abstraction were defined by a prospective protocol. We searched PubMed, and the Cochrane Collaboration Database through April 2006, using the following search terms: 'hemoperfusion or hemadsorption or hemodiafiltration or hemofiltration or hemodialysis' and 'polymyxin or polymyxin B or Toraymyxin or PMX-DHP or DHP-PMX' without language restrictions. We also reviewed bibliographies of retrieved articles and consulted with experts to identify relevant studies. Other methods of study identification included searching names of authors of relevant studies and contacting industry. Published English, Japanese, and Italian language full-text case series, cohort studies, and RCTs of DHP-PMX were eligible. Japanese articles were translated by a competent scientific/medical translator with a knowledge of the subject matter. To further facilitate translation, the translator was given instructions regarding the specific data being abstracted as well as specific statements of interest to the reviewers (for example, regarding randomization, blinding, and follow-up).

Prospective and retrospective observational studies, pre- and post-intervention design, and RCTs reporting original data on five or more adults treated with PMX-F for sepsis were included. Three authors reviewed all citations and abstracted data independently on a standardized form, and disagreements were resolved by discussion. Included trials had at least one of the following outcome measures: mean arterial pressure (MAP), doses of vasoactive agents, arterial partial pressure of oxygen/fraction of inspired oxygen (PaO_2_/FiO_2_) ratios, endotoxin levels, and mortality. We contacted authors and invited them to provide data for inclusion in the meta-analysis if we were unable to extract data directly from the publication or when relevant data were presented only in graphical form or only as subgroups (for example, survivors and non-survivors, by levels of Acute Physiology and Chronic Health Evaluation [APACHE] score). If the authors did not provide the data, these studies were excluded.

If multiple publications by the same investigator existed, the studies were reviewed carefully and/or the investigator was contacted to ensure that no data were analyzed in duplicate. At least three attempts were made to contact the corresponding and/or first investigator. Methods included e-mail and mailed letters. Three investigators independently assessed trial quality with the validated scale by Jadad and colleagues [26], which measures blinding, randomization, withdrawals, and dropouts. A maximum score of 5 represents the highest quality trial.

The primary endpoints were change in MAP, use of vasoactive agents and PaO_2_/FiO_2 _ratio at the end of DHP-PMX, and mortality. A secondary endpoint was the change in endotoxin levels after DHP-PMX. Assuming a standard deviation of 20 mm Hg for MAP pre- and post-PMX-F, a sample size of at least 70 patients would be needed to detect a change in MAP of at least 10 mm Hg in a paired analysis. For continuous variables such as blood pressure, data in the published studies generally were presented as a pooled summary of pre-PMX-F treatment versus post-PMX-F treatment rather than PMX-F versus conventional therapy. In many of the parallel studies, 'post-conventional therapy' values were not reported for this group. Therefore, for continuous outcomes, the effect size was the change (follow-up minus baseline) for each parameter in patients treated with PMX-F. The 'post-PMX-F' values used for the analyses were those 24 to 48 hours after the last PMX-F treatment. We combined data from parallel-designed trials with those from 'pre-post' studies in a meta-analysis using the generic inverse variance method. In both types of studies, we recorded the mean change from baseline values for the PMX group and variance estimates for this change, when reported. When these were not reported, we attempted to obtain these values or paired individual data directly from the authors. Not all investigators provided the information requested. For the studies in which these data were not available, we calculated these values as the difference between the mean 'pre-PMX-F' and 'post-PMX-F' values, and their variance estimates were derived from confidence intervals (CIs), standard deviations, and probability values reported in the manuscript [27]. Among the studies in which 'pre-PMX-F,' 'post-PMX-F,' and change variance estimates were available, the median correlation between the two periods was 0.59 (range, 0.05 to 0.93). To be conservative, we assumed a correlation of 0.5 to impute missing change variance estimates in the primary analysis. We performed sensitivity analyses of this choice of correlation, using 0.05 as the most conservative estimate, and the results remained robust. With regard to the endpoint of mortality, because DHP-PMX is an invasive and costly procedure, we considered it acceptable as a treatment for sepsis if a 15% absolute risk reduction could be achieved. Assuming a 50% mortality in the conventional medical therapy group, an α of 0.05, and 80% power, a sample size of at least 182 subjects in each arm is needed for parallel studies. Studies were considered for inclusion in the mortality analysis if they reported mortality for a comparable patient group (for example, sepsis) in the ICU which was not treated with PMX-F. Death was determined at the end of follow-up (14 to 60 days), as available. Results for mortality were combined on the risk ratio (RR) scale. Because the random effects model incorporates statistical heterogeneity and provides a more conservative estimate of the pooled effect size than a fixed model, we present the results of all analyses according to a random model. Intertrial heterogeneity was estimated by chi-square test. Sensitivity analyses were predefined *a priori *to evaluate the effects of study design, sample size, type of infection (gram-positive or -negative), imputed values for the correlation coefficient (discussed above), and center duplication. Because some investigators had more than one publication, for each endpoint we performed a sensitivity analysis in which we included only one study per investigator group, selecting the study with the largest sample size. We also assumed that the magnitude of change in certain clinical parameters would be dependent on the baseline value and performed a sensitivity analysis based on baseline blood pressure, PaO_2_/FiO_2 _ratio, and endotoxin levels. Funnel plots were drawn to examine whether the smaller studies in the meta-analysis tended to show larger treatment effects, which might be due to publication bias.

Analyses were performed with Review Manager version 4.2 (RevMan; The Cochrane Collaboration 2003, Nordic Cochrane Centre, Copenhagen, Denmark). The level of statistical significance is set at a *P *value of less than 0.05. For continuous outcomes, the changes in the parameter (for example, MAP) are expressed in their original linear scale as a point estimate with 95% CIs and *P *value. For mortality, values for RR are expressed as a point estimate with 95% CIs and *P *value. All RRs refer to the risk for the PMX group compared with the conventional medical therapy group (labeled in graphs as 'PMX' and 'Conventional,' respectively).

## Results

### Identification of eligible trials

**Figure 1:**
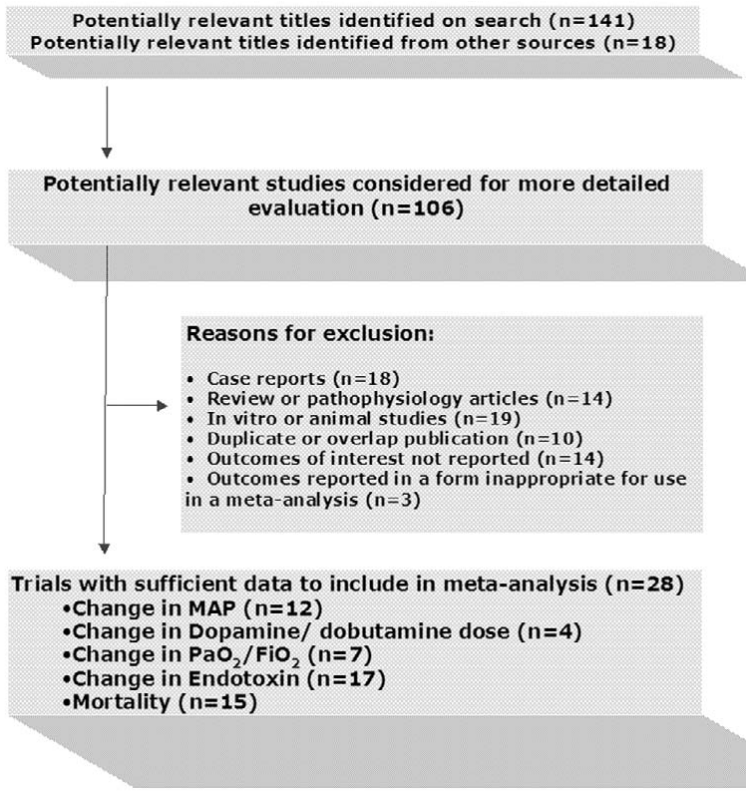
Details of included and excluded trials. MAP, mean arterial pressure; PaO_2_/FiO_2_, arterial partial pressure of oxygen/fraction of inspired oxygen.

One hundred fifty-nine abstracts were reviewed. Of these, 106 articles were deemed worthy of further exploration and review (Figure 1). Potentially relevant Japanese articles were translated to assess for inclusion. On careful review and confirmation with authors, all were found to have patient overlap with subsequent publications by the same authors in English language journals and were excluded for this reason. We identified a total of 28 publications as relevant to this review (Tables 1 and 2). Of these, 16 parallel trials (9 RCT and 7 non-RCT) and 12 pre-post cohort studies reported at least one of the necessary outcome measures and were included in the analysis (pooled sample size for parallel studies = 1,040 [RCT = 474], for pre-post studies = 385).

**Table 1:** Characteristics of included studies: parallel-design studies

Study	Year	Country of origin	Randomization	Conventional therapy				PMX-F			
				*N*	Percentage of males	APACHE II score	Predicted mortality (percentage)	*N*	Percentage of males	APACHE II score	Predicted mortality (percentage)
Tani *et al*. [21] (a)^a^	1998	Japan	No	33	69.7	SSS 39.1	N/A	37	78.4	SSS 46.2	N/A
Nakamura *et al*. [10]	1999	Japan	Yes	20	60.0	NS	N/A	30	60.0	24.8	52.6
Nemoto *et al*. [14]	2001	Japan	Yes	44	61.4	23	46.0	54	64.7	22	42.4
Nakamura *et al*. [11] (a)	2002	Japan	Yes	9	66.7	27.5	62.2	9	66.7	28.5	65.6
Suzuki *et al*. [15]	2002	Japan	Yes	24	70.8	25	53.3	24	75.0	25	53.3
Tsushima *et al*. [33]	2002	Japan	No	10	80.0	NS	N/A	24	70.8	22.4	43.9
Tsugawa *et al*. [31]	2002	Japan	No	51	43.1	NS	N/A	31	45.2	NS	N/A
Nakamura *et al*. [8] (b)	2003	Japan	Yes	10	60.0	27	60.5	10	60.0	27.6	62.5
Nakamura *et al*. [12] (c)	2003	Japan	Yes	25	64.0	23	46.4	35	68.6	24.2	50.4
Nakamura *et al*. [29] (d)	2003	Japan	No	108	62.0	24	49.7	206	64.1	24.6	51.9
Nakamura *et al*. [13] (e)	2004	Japan	Yes	10	60.0	28	63.9	15	60.0	28.4	65.2
Nakamura *et al*. [9] (f)	2004	Japan	Yes	50	64.0	24.8	52.6	70	61.4	25.4	54.8
Ono *et al*. [30]	2004	Japan	No	13	61.5	8.8	9.7	10	60.0	19.6	34.2
Tsujimoto *et al*. [32]	2004	Japan	No	10	20.0	10.6	12.2	7	85.7	19.4	33.5
Nakamura *et al*. [19] (g)	2005	Japan	No	12	58.3	25	53.3	14	64.3	25.5	55.1
Vincent *et al*. [28]	2005	Belgium, UK, Germany, Netherlands, Spain	Yes	18	47.4	18.7	31.3	17	76.5	16.7	25.4
Total				447				593			

^a^Two studies reported severity of illness as SSS rather than APACHE score.

APACHE II score expressed as the mean. Predicted mortality was calculated as e^Logit^/(1+ e^Logit^), where Logit = -3.517 + (APACHE II) × 0.146. APACHE, Acute Physiology and Chronic Health Evaluation; N/A, not applicable; NS, not stated; PMX-F, polymyxin B-immobilized fiber column; SSS, Sepsis Severity Score.

**Table 2:** Characteristics of included studies: pre-post design studies

Study	Year	Country of origin	*N*	Percentage of males	APACHE II score	Predicted mortality (percentage)
Nakamura *et al*. [16] (h)	1998	Japan	24	58.3	26.8	59.8
Nakamura *et al*. [17] (i)	1998	Japan	17	58.8	23.1	46.4
Shimada *et al*. [20]	2000	Japan	40	NS	NS	N/A
Tani *et al*. [36] (b)	2001	Japan	88	71.6	24.2	50.4
Uriu *et al*. [24]	2002	Japan	24	66.7	NS	N/A
Ikeda *et al*. [34]	2004	Japan	66	NS	26.2	57.6
Nakamura *et al*. [18] (j)	2004	Japan	12	66.7	24.5	51.5
Tojimbara *et al*. [22]	2004	Japan	24	45.8	21.4	40.3
Kushi *et al*. [35]	2005	Japan	36	58.3	24	49.7
Ueno *et al*. [23]^a^	2005	Japan	16	31.3	SSS 32	N/A
Kojika *et al*. [37]	2006	Japan	24	62.5	14.2	19.1
Casella *et al*. [38]	2006	Italy	14	57.1	26.5	58.7
Total			385			

^a^Two studies reported severity of illness as SSS rather than APACHE score.

APACHE II score expressed as the mean. Predicted mortality was calculated as e^Logit^/(1 + e^Logit^), where Logit = -3.517 + (APACHE II) × 0.146. APACHE, Acute Physiology and Chronic Health Evaluation; N/A, not applicable; NS, not stated; SSS, Sepsis Severity Score.

### Methodological quality of included studies

Three independent reviewers allocated a score of methodological quality. There was no disagreement between reviewers in any case. Overall, study quality was poor (Jadad scores of less than 3). Among the randomized studies, allocation concealment was deemed adequate in three trials [8,9,28] and uncertain in six [10-15]. Randomization was not performed in seven of the parallel-design studies [19,21,29-33]. Like most trials on extracorporeal therapies, none of the studies was double-blinded. Although very few studies had a specific statement on loss of follow-up (which merits 1 point on the Jadad scale), it was generally clear from the presented data that all patients were accounted for in terms of mortality in these short-term studies.

### Characteristics of patients and interventions

The 28 trials included 1,425 patients: 978 in the PMX-F group and 447 in the conventional medical therapy group. Of these, 26 studies reported the mean age of the patients (range, 39 to 78.5 years), 26 reported the proportion of men (range, 20% to 85.7%), and 23 reported the baseline severity of illness at the time of enrollment as APACHE II score (range, 8.8 to 28.5 points). Characteristics of the included studies are shown in Tables 1 and 2. Two RCTs enrolled patients with methicillin-resistant *S. aureus *(MRSA) infections [8,12]. When reported, gram-negative infections were identified in 71% of patients (range, 37.9% to 100% in individual studies) [10,11,13-17,21-24,28-30,32,34-38].

DHP-PMX was performed with an adsorbent column that was designed for clinical use and that contained 5 mg of PMX per gram of polystyrene fiber and with a priming volume of 135 ml (Toray Industries, Inc., Tokyo, Japan) [7,39]. The usual indication for DHP-PMX was sepsis (with or without septic shock) as defined by the American College of Chest Physicians/Society of Critical Care Medicine Consensus Conference Committee [40]. DHP-PMX was performed for two hours at a blood flow rate of 50 to 150 ml/minute, once [15,21,28,30-32,34] or a maximum of two [8,9,11-14,16-20,22-24,29,33,35] or three [38] times, depending on the clinical response of the patient. When necessary, the succeeding PMX-F treatment was performed 24 hours after the previous treatment. Nafamostat mesilate and unfractionated or low-molecular-weight heparin were used as the anticoagulant [13-16,18,21-24,28,29,33-36,38]. The type of anticoagulant was not specified in other studies [8-12,17,19,20,30-32,37]. DHP-PMX was performed in addition to conventional medical therapy, which included antibiotic therapy, administration of gamma-globulins, vasopressors, hemodynamic monitoring, organ support in the ICU including mechanical ventilation, corrective measures for metabolic abnormalities [8-15,23,29,35,38], and surgery when appropriate [28,31]. In five studies, renal replacement therapy [9,15,22,23,38] was also performed for renal failure. One study specifically enrolled patients with acute renal failure [15] and another, chronic renal failure [22].

### Effects on MAP and dose of vasoactive agents

**Figure 2:**
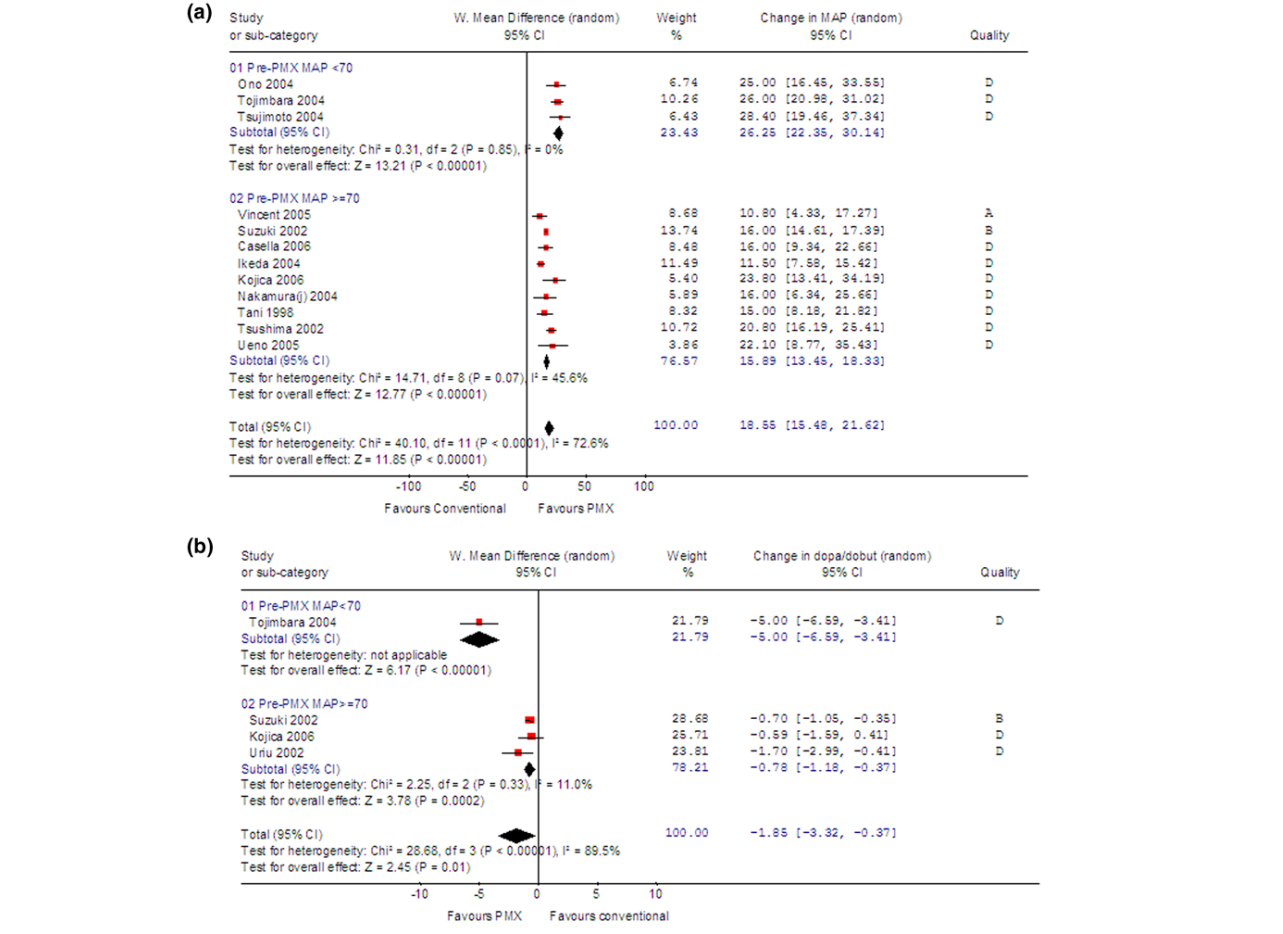
**(a) **Change in MAP after PMX-F (275 participants included). **(b) **Change in dopamine/dobutamine dose after PMX-F (96 participants included). CI, confidence interval; MAP, mean arterial pressure.

The effect of PMX-F therapy on MAP was ascertained in a pooled analysis of 12 studies (2 RCT, 4 non-RCT, and 6 pre-post; 275 patients) [15,18,21-23,28,30,32-34,37,38]. The method of measuring blood pressure was not specified in any of the articles. All studies that provided sufficient data reported improvement in MAP after PMX-F (Figure 2a). The pooled estimate showed that PMX-F was associated with a significant increase in MAP (weighted mean difference, 19 mm Hg; 95% CI, 15 to 22 mm Hg; *p *< 0.001). This represented a 26% mean increase in MAP (range, 14% to 42%). However, intertrial heterogeneity in this primary analysis was significant (*p *< 0.001). Because the magnitude of change in blood pressure would be dependent on the baseline value, subgroup analysis was performed based on the mean pre-PMX MAP in the PMX-F group (Figure 2a). Patients with a mean pre-PMX MAP below 70 mm Hg had a greater improvement in MAP (26 mm Hg) compared to those with a mean pre-PMX MAP of at least 70 mm Hg (16 mm Hg). Selected sensitivity analyses are shown in Table 3. Intertrial heterogeneity became non-significant when the analysis was limited to subgroups defined by pre-PMX MAP; however, there was still substantial heterogeneity (45.6%) in the subgroup with greater than or equal to 70 mm Hg (Figure 2a).

**Table 3:** Selected sensitivity analysis

	No. of studies	No. of patients	Effect size	95% CI	Overall effect (*P *value)	Heterogeneity (*P *value)
Change in MAP			(mm Hg)			
All	12	275	19	(15, 22)	< 0.001	< 0.001
*n *> 20	5	175	18	(13, 22)	< 0.001	< 0.001
Pre-PMX MAP < 70	3	41	26	(22, 30)	< 0.001	0.85
Pre-PMX MAP ≥ 70	9	234	16	(13, 18)	< 0.001	0.07
Center duplication	11	268	18	(15, 21)	< 0.001	< 0.001
						
Change in dopamine/dobutamine dose			(μg/kg per minute)			
All^a^	4	96	-1.8	(-3.3, -0.4)	0.01	< 0.001
Pre-PMX MAP <70	1	24	-5.0	(-6.6, -3.4)	< 0.001	N/A
Pre-PMX MAP ≥ 70	3	72	-0.8	(-1.2, -0.4)	< 0.001	0.33
						
Change in PaO_2_/FiO_2 _ratio			(Units)			
All^a^	7	151	32	(23, 41)	< 0.001	0.87
Pre-PMX PaO_2_/FiO_2 _ratio < 200	2	36	30	(19, 40)	< 0.001	0.62
Pre-PMX PaO_2_/FiO_2 _ratio ≥200	5	115	40	(20, 60)	< 0.001	0.84
						
Change in endotoxin level			(pg/ml)			
All	17	455	-21.2	(-24.9, -17.5)	< 0.001	< 0.001
Excluding MRSA	15	410	-24.1	(-28.0, -20.2)	< 0.001	< 0.001
Pre-PMX endotoxin <30 pg/ml	5	97	-9.8	(-12.1, -7.5)	< 0.001	0.007
Pre-PMX endotoxin ≥30 pg/ml	12	358	-28.2	(-32.3, -24.1)	< 0.001	< 0.001
Pre-PMX endotoxin <40 pg/ml	10	283	-14.9	(-18.7, -11.1)	< 0.001	< 0.001
Pre-PMX endotoxin ≥40 pg/ml	7	172	-37.4	(-41.9, -32.8)	< 0.001	0.25
Center duplication	6	225	-16.4	(-24.0, -8.9)	< 0.001	< 0.001
						
Mortality			Risk ratio			
All	15	920	0.53	(0.43, 0.65)	< 0.001	0.07
RCT	8	354	0.50	(0.37, 0.68)	< 0.001	0.12
Parallel non-RCT	7	566	0.55	(0.38, 0.81)	0.002	0.07
Excluding MRSA	13	840	0.55	(0.44, 0.69)	< 0.001	0.08
RCT excluding MRSA	6	274	0.55	(0.40, 0.76)	< 0.001	0.2
*n *> 20	7	722	0.56	(0.46, 0.68)	< 0.001	0.17
APACHE II score < 25	9	713	0.56	(0.43, 0.73)	< 0.001	0.06
APACHE II score ≥ 25	6	207	0.45	(0.30, 0.68)	< 0.001	0.25
28- to 30-day mortality only	9	704	0.54	(0.43, 0.68)	< 0.001	0.12
Center duplication	8	673	0.61	(0.46, 0.82)	0.001	0.03

^a^For outcomes of dopamine/dobutamine dose and PaO_2_/FiO_2 _ratio, all included studies had a sample size greater than 20. APACHE, Acute Physiology and Chronic Health Evaluation; CI, confidence interval; MAP, mean arterial pressure; MRSA, methicillin-resistant *Staphylococcus aureus*; N/A, not applicable; PaO_2_/FiO_2_, arterial partial pressure of oxygen/fraction of inspired oxygen; PMX, polymyxin B-immobilized fiber column; RCT, randomized controlled trial.

In critically ill patients, it is often difficult to interpret blood pressure in isolation because vasoactive agents can be manipulated to alter the blood pressure. In four studies, the dose of dopamine or dobutamine or the average of the sum of the two was reported [15,22,24,37]. All studies showed a trend toward a decrease in the dose after PMX-F (Figure 2b). Overall, the dose was decreased by 1.8 μg/kg per minute (95% CI, 0.4 to 3.3 μg/kg per minute; *p *= 0.01). In these studies, there was also an increase in mean MAP after PMX-F (range, 16 to 28 mm Hg).

### Effects on PaO_2_/FiO_2 _ratio

The effect of PMX-F therapy on PaO_2_/FiO_2 _was ascertained in a pooled analysis of seven studies [18,22,28,33,35,37,38] (151 patients), only one of which was an RCT [28]. Overall, the PaO_2_/FiO_2 _ratio increased by 32 units (95% CI, 23 to 41 units; *p *< 0.001) after PMX-F. Intertrial heterogeneity was not significant. In the single RCT in this analysis [28], there was a non-significant trend toward an improvement in PaO_2_/FiO_2 _ratio in the PMX-F group (delta PaO_2_/FiO_2 _ratio 29.54 versus 0.43 units in the PMX-F and control groups, respectively; *p *= not significant).

### Effects on mortality

**Figure 3:**
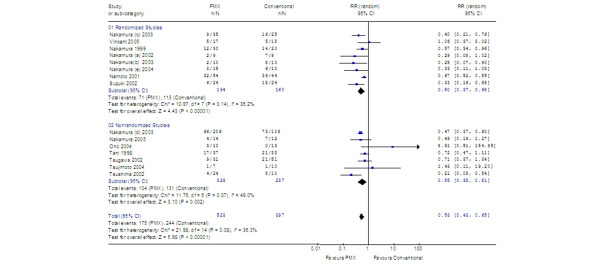
Risk ratio (RR) for death after polymyxin B-immobilized fiber column (PMX-F) treatment (920 participants included in meta-analysis). CI, confidence interval.

**Figure 4:**
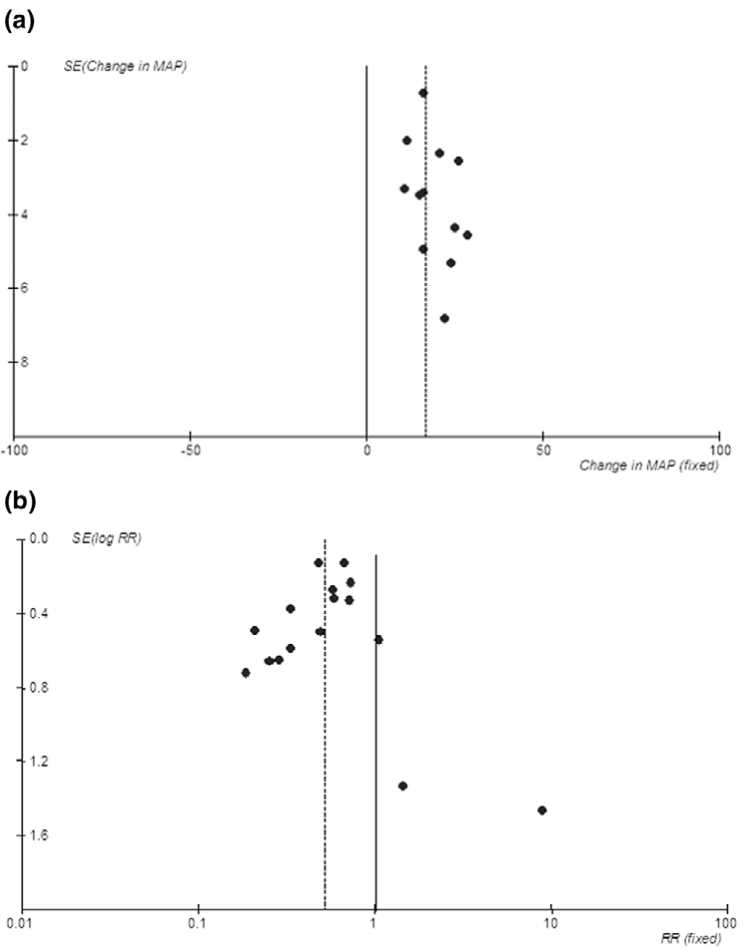
Funnel plots. **(a) **Mean arterial pressure (MAP). **(b) **Mortality. Solid line represents line of neutral effect. Dashed line represents point estimate of analysis. RR, risk ratio; SE, standard error.

Data on mortality, variably reported as 14-day [21], 28-day [14,15,19,28,29,32], 30-day [10,31,33], and 60-day [12], were available from 11 studies. Mortality was reported but length of follow-up was not clearly stated in another four studies [8,11,13,30] (for a total of 15 studies: 8 RCT and 7 non-RCT; 920 patients). One study had sepsis patients in the conventional therapy group and septic shock patients in the PMX-F group [30]. Pooled mortality rates were 61.5% in the conventional therapy group and 33.5% in the PMX-F group. In the pooled estimate, PMX appeared to significantly reduce mortality compared with conventional medical therapy (Figure 3) (RR, 0.53; 95% CI, 0.43 to 0.65). The results were similar between RCT and non-RCT and when two RCTs enrolling patients with MRSA infections were excluded (RR, 0.55; 95% CI, 0.40 to 0.76; Table 3). When the analysis was limited to the nine studies that reported 28- to 30-day mortality [10,14,15,19,28,29,31-33], results were unchanged. Various sensitivity analyses are shown in Table 3. The funnels plots of standard error against effect size for MAP and mortality are shown in Figure 4. For MAP (Figure 4a), the effect size of RCTs was smaller than in non-randomized parallel or pre-post studies. For mortality (Figure 4b), three small studies (*n *= 17 to 35) had point estimates for RR of greater than 1.

### Effects on endotoxin levels

In 19 studies (9 RCTs, 2 parallel non-RCTs, and 8 pre-post), data on endotoxin levels were available [8-24,28,37]. In 17 of these [8-24], endotoxin levels were measured by the Endospecy method (upper limit of normal is 9.8 pg/ml; Seikagaku Corporation, Tokyo, Japan) [39]. In one study [28], endotoxin was measured with the modified limulus amebocyte lysate (LAL) assay by means of a commercial kit (COATEST; DiaPharma Group, Inc., West Chester, OH, USA), which is sensitive up to 0.05 endotoxin units [EU]/ml in serum or plasma. In another [37], it was measured by kinetic turbidimetric limulus assay by means of MT-251 Toxinometer (Wako Pure Chemicals Industries, Ltd., Osaka, Japan), which is sensitive up to 0.01 pg/ml. For consistency, we limited our analysis to the studies that used the Endospecy method (*n *= 17 studies, 455 patients). In one of these studies, the authors reported endotoxin levels only for patients with gram-negative infections [22]. The pooled estimate showed that endotoxin levels decreased by 21.2 pg/ml (95% CI, 17.5 to 24.9 pg/ml) after PMX-F, representing a decrease of 33% to 80% from pre-PMX levels. Results were similar when the analysis excluded two RCTs [8,12] on MRSA infections (Table 3). There was significant intertrial heterogeneity for this outcome (*p *< 0.001). After exclusion of a trial with large standard deviation [21] in a *post hoc *sensitivity analysis, results were unchanged and intertrial heterogeneity remained significant (*p *< 0.001). Various sensitivity analyses, including stratification by pre-PMX endotoxin levels, did not qualitatively alter results (Table 3). As expected, the baseline endotoxin levels were lower in the two trials enrolling patients with MRSA infections [8,12]. To evaluate the effect of over-representation of two centers representing several of the 17 studies included in the meta-analysis, we also performed a sensitivity analysis in which we used only the study with the largest sample size from these centers [9,14], and the results were qualitatively similar. In the Japanese study which used the turbidimetric limulus assay, mean endotoxin levels decreased significantly from 8.84 to 2.11 pg/ml [37] after PMX-F, whereas in the European RCT in which endotoxin was measured with the modified LAL assay, endotoxin levels did not change significantly (median, 28 EU pre-PMX-F to 38.5 EU 24 hours post-PMX) [28].

### Adverse events

Only two studies reported adverse events, and these included clotting of the device in 4/21 filter cartridges [28] and hypersensitivity (erythema) in 2/35 patients [8]. Specifically, no adverse events indicative of nephrotoxicity (including cellular casts) or neurotoxicity (including irritability and progressive weakness) were reported in any of the studies. A third study stated that there were no adverse events related to DHP-PMX [38].

## Discussion

This systematic review of 28 published studies, which included more than 1,400 patients treated in seven countries, suggested multiple beneficial benefits of direct hemoperfusion with PMX-F as compared with conventional medical therapy for patients with sepsis and septic shock. The positive effects are thought to be due to reductions in endotoxin levels by DHP-PMX. Both the data from individual studies and the combined data suggest that PMX-F increases blood pressure, reduces use of vasoactive agents, and lowers endotoxin levels as measured by the Endospecy method. The pooled estimate also suggests that PMX-F improves gas exchange, as represented by the PaO_2_/FiO_2 _ratio, although the single RCT that reported this outcome showed only a non-significant positive trend [28]. In addition, there appeared to be an appreciable effect on mortality. However, these findings must be interpreted with caution because overall study quality was suboptimal and very few of these studies were planned or powered to specifically assess mortality. The invasive nature of DHP-PMX-F makes an absolute risk reduction of mortality by 15% acceptable. This systematic review had adequate power to detect a risk reduction of this magnitude.

Our analysis of hemodynamic effects of PMX-F was limited to MAP and vasopressor use. Individual studies have reported improvement in systemic vascular resistance [18,21,24,41,42], cardiac output (CO), or cardiac index [24,42] in certain patient subgroups. Unfortunately, there were insufficient pooled data on these outcomes for meta-analysis. Nevertheless, pooled data seem to indicate that PMX-F therapy increases blood pressure while simultaneously reducing the dose of vasoactive agents [15,22,24,37], strongly suggesting a clinically significant improvement in hemodynamic status.

We cannot conclude from our analysis whether the beneficial clinical effects are directly related to removal of endotoxin or perhaps other substances in the circulation. Reduced levels of other mediators such as IL-6 [15,30,36], IL-10 [30,36], IL-18 [10], TNF-α [34,36], metalloproteinase-9 [18], plasminogen activator inhibitor-1 [34-36,43], neutrophil elastase [35,44], platelet factor-4 [10], β-thromboglobulin [10], soluble P selectin [10], and endogenous cannabinoids [45] such as anandamide have also been reported after DHP-PMX. Work by Uriu and colleagues [24] suggests that reduction in blood endotoxin concentration by PMX-F therapy positively correlated with the reduction in CO, regardless of the causative organism.

Potential adverse events with this treatment include thrombocytopenia and hypotension during DHP-PMX as well as the known nephrotoxic and neurotoxic effects of polymyxin B. The latter two are theoretically avoided because the polymyxin B is not released into the circulating blood. Very few adverse events were reported among the included publications, suggesting that the treatment is generally well tolerated. However, the authors concede that small sample sizes would greatly impair the ability to observe rare but significant adverse effects.

A major potential source of bias in systematic reviews is that trials with positive results are more likely to be published than trials with negative or neutral results. In Japan, where PMX-F has been available for more than 10 years, the results of only a fraction of the patients treated with PMX-F have been published. The results of any review are also inherently limited by the quality of the primary studies. For the outcomes of dopamine/dobutamine dose and PaO_2_/FiO_2 _ratio, there was only a single RCT among the analyzed studies. To address this, we performed a limited number of predefined various sensitivity analyses (Table 3), and the overall effect sizes were still significant. Most reported comparisons in the literature were pre-treatment versus post-treatment in the PMX-F group only. We therefore chose to analyze continuous outcomes only within the PMX-F group rather than make a direct comparison between PMX-F versus conventional therapy. Other interventions performed in routine intensive care besides host and disease factors certainly change these variables, making the interpretation of the positive variation (as we have found) difficult to definitively ascribe to PMX-F. Although this is not the ideal method for determining the true effects of PMX-F, it was thought to adequately represent the published data. Nevertheless, this approach will always tend to show a bias toward improvement because the data will tend to over-represent the survivors, particularly in a high-mortality disease such as sepsis. The 'post-PMX-F' values used in the analysis were those obtained 24 to 48 hours after the last treatment. It is not clear whether deaths occurred predominantly within this short time frame or later. Therefore, the results of these analyses should be interpreted with caution, and prospective validation is needed before causal inferences can be made. Although it may be reasonable to question the wisdom, and indeed the validity, of mathematically combining results from such studies, the authors felt that such an attempt was warranted, at the very least to provide a crude estimate of the likely effect of PMX-F for the specified outcomes. The overall mortality of 61.5% observed in the conventional therapy group was comparable to values observed in a French multicenter study on moderate-dose corticosteroid therapy (63%) [46] and a Brazilian study on protective ventilation (71%) [47] and was higher than those reported in studies on early goal-directed therapy (46.5%) [48] and activated protein-C (30.8%) [49]. Moreover, mortality in the conventional therapy group within the various studies averaged 58% (range, 0% to 88.6%), which was higher than the predicted mortality based on APACHE II scores (mean, 45.1%; range, 9.7% to 63.9%). Another potential issue is that of multiple publication bias. However, in addition to carefully reviewing the articles, we made every effort to contact authors about this and were able to confirm with two sets of authors with multiple publications. We also performed sensitivity analyses to estimate the effect of over-representation of data from centers with several publications. Nevertheless, all but two of the included studies come from 12 groups in Japan, and the generalizability of these findings ultimately will require further study. Only one RCT has been performed outside Japan [28], and its findings are in contrast with those performed in Japan (Figure 3), highlighting concerns about the generalizability and reproducibility of such studies outside this setting. It is worth noting, however, that this European RCT enrolled patients with lower APACHE scores (Table 1) compared to the Japanese RCTs and had a relatively small sample size. Although it is acknowledged that patient and physician blinding is difficult to achieve in controlled studies of extracorporeal therapies, it was generally not stated in any of the studies whether the data analyst was blinded to the groupings. As with several studies in the critical care arena, the focus of publications is usually on short-term outcomes, and it is impossible to comment on the long-term effects of PMX-F. Lastly, the available data do not allow us to comment on the optimal timing for PMX-F therapy.

## Conclusion

Putting these data into perspective, this systematic review of the published literature found positive effects of PMX-F on MAP and dopamine/dobutamine use, PaO_2_/FiO_2 _ratio, endotoxin removal, and mortality. Overall, however, the analyzed studies were of suboptimal quality, which may exaggerate the magnitude of these effects. These putative benefits remain to be determined definitively in a prospective trial with appropriate clinical endpoints. Because there seemed to be beneficial effects even among patients with gram-positive infections such as MRSA, further investigations on this group of patients and those with mixed infections would be worthwhile. With the recent availability of commercial kits that are able to accurately measure endotoxin in blood [2], trial enrollment on the basis of endotoxin level rather than a documented type of organism or presence of clinical signs of septic shock may allow investigators to better evaluate the effects of early treatment with PMX-F. Certainly, additional work on the optimal timing of PMX-F treatment is needed. Our analysis of PaO_2_/FiO_2 _ratio also suggests a possible benefit in adult respiratory distress syndrome or acute lung injury and a need for further studies in this regard. The effect of this therapy on the development of acute kidney injury and the need for renal replacement therapy, which is associated with a high mortality, is another area for further study.

## Key messages

• Polymyxin B binds endotoxin, one of the principal biological substances that cause gram-negative septic shock, but has adverse nephrotoxic and neurotoxic effects.

• DHP-PMX would theoretically allow removal of circulating endotoxin without systemic side effects.

• Based on published literature, DHP-PMX appears to effectively reduce endotoxin levels and have some positive effects on blood pressure, use of vasoactive agents, gas exchange, and mortality.

• These putative benefits have to be confirmed in adequately powered prospective trials.

## Abbreviations

APACHE = Acute Physiology and Chronic Health Evaluation; CI = confidence interval; CO = cardiac output; DHP-PMX = direct hemoperfusion with polymyxin B-immobilized fiber column; EU = endotoxin units; ICU = intensive care unit; IL = interleukin; LAL = limulus amebocyte lysate; MAP = mean arterial pressure; MRSA = methicillin-resistant *Staphylococcus aureus*; PaO_2_/FiO_2 _= arterial partial pressure of oxygen/fraction of inspired oxygen; PMX-F = polymyxin B-immobilized fiber column; RCT = randomized controlled trial; RR = risk ratio; TNF-α = tumor necrosis factor-alpha.

## Competing interests

The authors declare that they have no competing interests.

## Authors' contributions

DNC designed the study, performed the data collection and review, performed the statistical analysis, and contributed to the writing of the paper. MAP designed the study, participated in the statistical analysis, and contributed to the writing of the paper. NP and VC performed the data collection and review and participated in the statistical analysis. RB and VMR helped in the design of the study and contributed to the writing of the paper. MdC, PL, and FN participated in the writing of the paper. TU participated in data collection. CR designed the study and participated in data interpretation. All authors read and approved the final manuscript.
